# Social mobilization and polarization can create volatility in COVID-19 pandemic control

**DOI:** 10.1007/s41109-021-00356-9

**Published:** 2021-02-11

**Authors:** Inho Hong, Alex Rutherford, Manuel Cebrian

**Affiliations:** Center for Humans and Machines, Max Planck Institute for Human Development, Lentzealle 94, 14195 Berlin, Germany

**Keywords:** Social mobilization, Political polarization, Social network, COVID-19

## Abstract

During the COVID-19 pandemic, political polarization has emerged as a significant threat that inhibits coordinated action of central and local institutions reducing the efficacy of non-pharmaceutical interventions (NPIs). Yet, it is not well-understood to what extent polarization can affect grass-roots, voluntary social mobilization targeted at mitigating the pandemic spread. Here, we propose a polarized mobilization model amidst the pandemic for demonstrating the differential responses to COVID-19 as mediated by the USA’s political landscape. We use a novel dataset and models from time-critical social mobilization competitions, voting records, and a high-resolution county-wise friendship network. Our simulations show that a higher degree of polarization impedes the overall spread of mobilization and leads to a highly-heterogeneous impact among states. Our hypothetical compliance campaign to mitigate COVID-19 spread predicts grass-roots mitigation strategies’ success before the dates of actual lockdowns in identically polarized states with more than three times of success rate than oppositely polarized states. Finally, we analyze the coupling of social mobilization leading to unrest and the growth of COVID-19 infections. These findings highlight social mobilization as both a collective precautionary measure and a potential threat to countermeasures, together with a warning message that the emerging polarization can be a significant hurdle of NPIs relying on coordinated action.

## Introduction

While coordinated action of the public is strongly demanded to mitigate the outbreak of coronavirus disease 2019 (COVID-19) (Holtz et al. 2020), countries around the world are facing unexpected social, economic and cultural barriers (Van Bavel et al. 2020). Political polarization is one of these barriers that may incapacitate government measures (Desvars-Larrive et al. 2020) by distrusting policies from the opposing parties and circulating false information within echo chambers (Bakshy et al. 2015). Thus, understanding polarization on social networks would be essential for effective interventions and collective voluntary support to mitigate the pandemic.

Social mobilization that has featured in a series of open challenges in the last decade (Pickard et al. 2011; Tang et al. 2011; Cebrian et al. 2012; Rahwan et al. 2012; Rutherford et al. 2013, 2013; Stefanovitch et al. 2014; Alstott et al. 2014; Naroditskiy et al. 2014, 2012; Oishi et al. 2014; Wang et al. 2015; Chen et al. 2016; Epstein et al. 2019; Rutherford et al. 2020) provides a helpful framework to understand influence and polarization on social networks in response to epidemics. Social mobilization is a phenomenon in which large groups of people participate, often online, to collaborate on a common cause. Participants typically share social ties which are influential in deciding to join. Social mobilization has been used to describe the spread of social influence as a repeated recruitment process (i.e., mobilization) on social networks (Cebrian et al. 2012; Rutherford et al. 2013) due to its wide applicability and predictability for interdependent social phenomena such as political campaigns, health promotion (Sims et al. 2014) and viral marketing (Stephen and Lehmann 2016). During the COVID-19 pandemic, several new mobilization-driven phenomena have been observed such as compliance campaigns, anti-lockdown protests and the spread of misinformation (Van Bavel et al. 2020; Kim and Walker 2020; Han et al. 2020). In addition, former mobilization phenomena related to epidemics highlight mobilization as a framework to anticipate how the public may respond to a second epidemic wave (Bento et al. 2020; Benzell et al. 2020; Cruickshank and Carley 2020) and further Non-Pharmaceutical Interventions (NPIs) (Chinazzi et al. 2020; Kraemer et al. 2020; Aleta et al. 2020); for example, anti-vaccination movements might be a critical issue when vaccination begins (Johnson et al. 2020). These movements highlight social mobilization as a framework for understanding the efficacy of pandemic control.

Explaining mobilization during the pandemic requires consideration of political polarization as we have observed a significant political bias in many social movements related to NPIs; for example, anti-lockdown protests are mostly led by libertarians [31] and economic liberals [32]. This political bias of mobilization can cause volatility in its overall spread by limiting recruitments between oppositely polarized individuals (Rutherford et al. 2020). Specifically, the complexity of political homophily in friendship (Bakshy et al. 2015) and the heterogeneous political landscape may generate the complexity in the spread of mobilization across the country.

Although these polarized mobilizations can critically undermine the efficacy of NPIs, we still lack a model for estimating this spread and how it would be related to NPIs for pandemic control. Integrating social mobilization within social networks, political landscapes and polarized communications would provide a comprehensive model for the coordinated action of polarized individuals. Hypothetical scenarios on different political orientations simulated by this model would help policy makers to facilitate less volatile pandemic control, and encourage individuals to cooperate with the control.

Using a social mobilization model and a novel dataset from time-critical social mobilization competitions (Cebrian et al. 2012; Rutherford et al. 2013), voting records and a US county-wise friendship network (Bailey et al. 2018), we show how political polarization impedes social mobilization, and we further demonstrate the coupling of mobilization and epidemics. Our simulation of a hypothetical compliance campaign demonstrates how many states could have been closed before the actual date of lockdowns. In addition, we identify the increasing coupling between mobilization and confirmed cases of COVID-19 by states. Lastly, we provide several political insights: the risk of polarization, mobilization-bases early warning, unintended effects and methods for successful mobilization.

## Modelling mobilization on a polarized friendship network

### Data

The polarized friendship network is built upon several novel datasets: the county-to-county friendship weights, the voting records, and the demographics. The county-to-county friendship weights are given by the Facebook Social Connectedness Index (SCI) dataset (Bailey et al. 2018) which represents the normalized counts of friendship pairs between the entire US counties. The political landscape of US counties is made by the voting records in the 2016 US Presidential Election provided by The New York Times. The demographics of counties are obtained from the US Census Bureau. For the confirmed cases of COVID-19 by states, we used COVID-19 data in the United States from The New York Times, based on reports from state and local health agencies.

### Model

To simulate the polarized mobilization process during the pandemic, we built upon the polarized mobilization model (Rutherford et al. 2013, 2020) which was developed to model the FiftyNifty political recruitment campaign and to demonstrate social mobilization in open challenges more broadly. This mobilization model simulates a branching recruitment process that starts from a group of seeds and spreads through the polarized friendship network. As the friendship network is built upon the data of friendship pairs, we consider this friendship network as a ground-truth, and isolate the polarization effect in the mobilization process. Below we present the detailed procedures of the simulation including seeding, activation, recruitment and termination.

**Seeding**. The simulation starts from $N_{s}$ homogeneous seeds labeled as all Democratic or Republican to reproduce political mobilization. We employ two seeding methods to demonstrate mobilization from different scales.

- Seeding from a county: For the simulations in which the mobilization starts from a single county, the seeds are located in the most populated county of the state of interest.
- Nationwide seeding: For the simulations that seeding is nationwide, the seeds are distributed to every county in proportion to its Democratic or Republican population according to whether this mobilization is Democratic-oriented or Republican-oriented.

In general, we use $N_{s} = 50{,}000$ following the average size of Democratic or Republican population in a county. The activation time of each seed is determined by a log-normal distribution of a mean of 1.5 day and a standard deviation of 5.5 days following the original model (Iribarren and Moro 2009). At the same time, the number of friends to be recruited (i.e., the branching factor *k*) is assigned to each seed following a Harris discrete distribution (i.e., $P(k) = H_{ab}/(b + k^{a})$) with a power-law exponent $a = 2.1$ and the mean $\langle k \rangle = 0.9$, where $H_{ab}$ is a normalization factor to hold $\sum _{k}P(k) = 1$ (Rutherford et al. 2013, 2020). These seeds are inserted into a priority queue, and activated in the order of the earliest activation time.

**Fig. 1:**
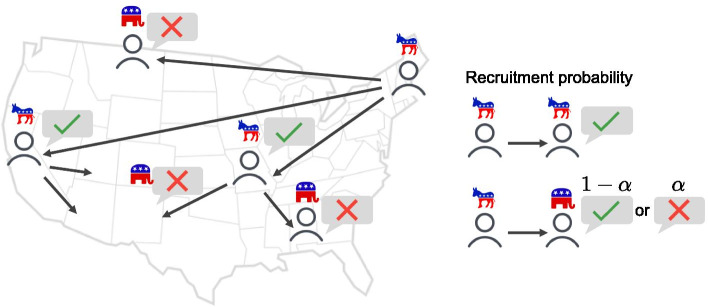
Illustration of the social mobilization process on a polarized friendship network. In our simulation, identically polarized friends always accept recruits, but oppositely polarized friends may reject a recruit with probability $\alpha$ which denotes the degree of polarization

**Activation**. Individuals in the queue are activated in the order of the earliest activation time in each simulation step. Mobilization of the activated person is determined by political polarization with its recruiter, and modulated by the polarization parameter $\alpha$ ranging from 0 to 1. When the activated person is oppositely polarized with its recruiter, mobilization has a lower success probability $p = 1-\alpha$, while mobilization for identical polarization is always successful as $p = 1$ (see Fig. 1). If the activated individual is a seed, it is mobilized with $p=1$.

**Fig. 2:**
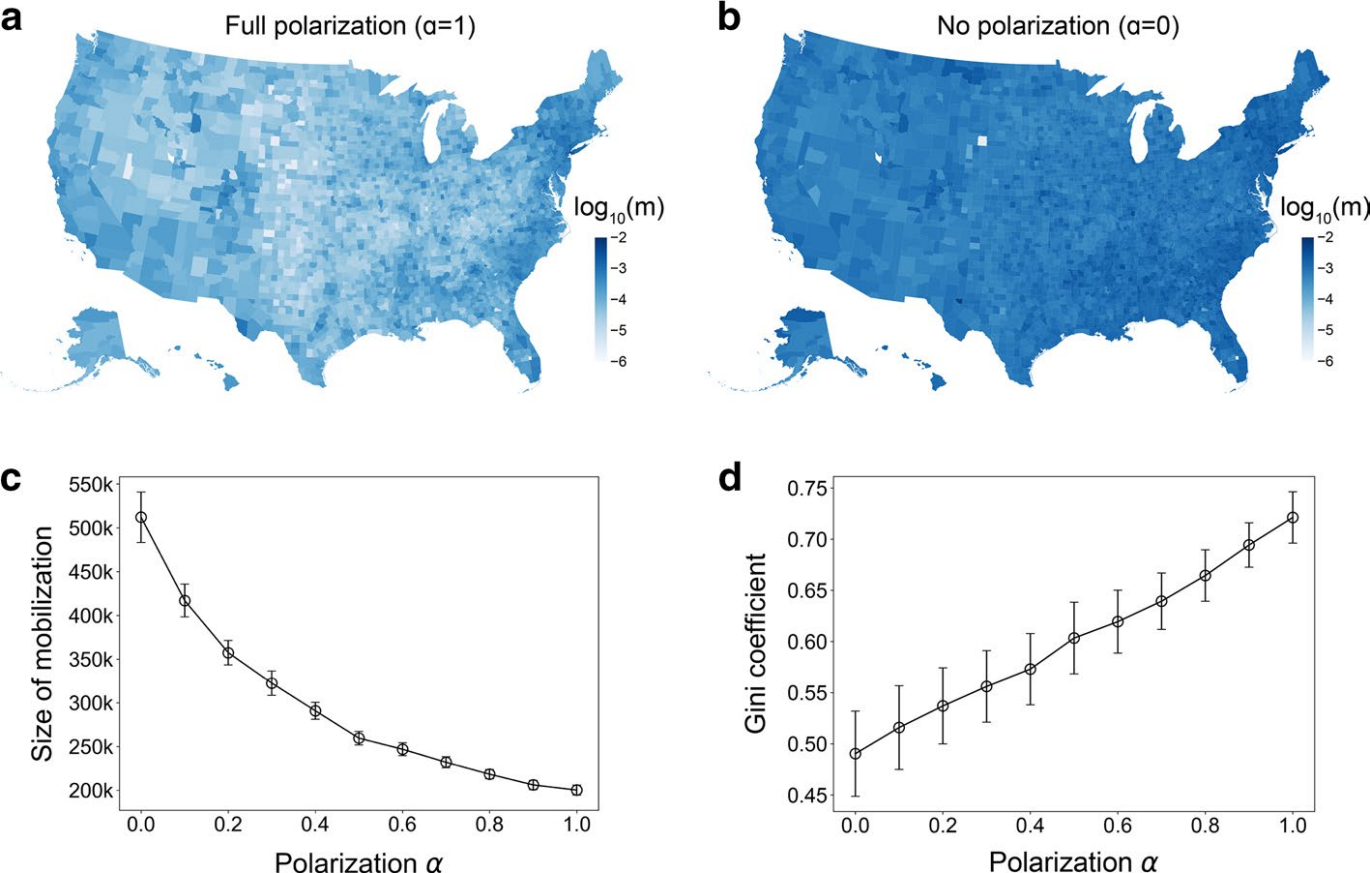
Size of mobilization as a function of political polarization. **a**, **b** Mobilizability of each county in the simulations for full polarization (**a**) and no polarization (**b**). We examined 100 simulations seeded from 50,000 Democratic populations in New York City. **c** The total size of mobilized individuals across the US for each polarization parameter ranging from 0 to 1. The plot is for the mean of 100 simulations and the error at the 95% percentile. **d** The heterogeneity of mobilizability measured by Gini coefficient for different levels of polarization. We measure the Gini coefficient of mobilizability of counties in each of 100 simulations, and take the average. The error bars denote the standard deviations

**Recruitment**. If this mobilization is successful, the mobilized person continues the recruitment process. The number of friends to be recruited is chosen by the identical branching factor distribution in **Seeding**. Likewise, their activation times are also determined by the identical log-normal distribution. The location of a recruited person is given by the friendship weights $SCI_{ij}$ from county *i* to county *j* in the Facebook SCI dataset. The probability that a recruiter in county *i* recruits a friend in county *j* is given as $p_{ij}=SCI_{ij}/\sum _{j}{SCI_{ij}}$ which is equal to the proportion of the friendship weight from *i* to *j* to the out-strength of *i*. Last, the political orientation of the recruited person is also stochastically determined in proportion to the political makeup of the county of residence and the homophilic political bias in friendship (Bakshy et al. 2015). More precisely, the probability is given as $(p_{dem}, p_{rep}) \sim (\frac{3}{4}pol_{c}, \frac{1}{4}(1-pol_{c}))$ for a Democratic recruiter and $(p_{dem}, p_{rep}) \sim (\frac{1}{4}pol_{c}, \frac{3}{4}(1-pol_{c}))$ for a Republican recruiter where $p_{dem}$ and $p_{rep}$ denote the probabilities of being Democratic and Republican, and $pol_{c}$ denotes the fraction of votes to the Democratic party in county *c*. The weights (3/4, 1/4) represent stronger social ties to identical polarization (Bakshy et al. 2015).

Unlike the original model (Rutherford et al. 2020), this seeding method involves recruitment within the same state (i.e., *i* = *j*) to better demonstrate mobilization during the pandemic. As the goal of the social challenge (i.e., the FiftyNifty challenge) was to mobilize friends in every state, mobilization in the original model spreads without mobilizing the same state, and terminates when at least one person is mobilized in every state. On the contrary, recruitment within the same state is allowed in this paper to mobilize as many people as possible no matter where they live.

**Termination**. The simulation is terminated when every state has at least 100 mobilized individuals or there is no remaining recruiter in the queue, i.e., the branching process terminates due to attrition.

## Mobilization impeded by polarization

To examine the effect of polarization on mobilization, we demonstrate the size of mobilization in each state using a simulation seeded from 50,000 Democratic populations in New York City. As the mobilization size of a region is intuitively proportional to its population size, we define “mobilizability” $m_{i}$ of region (i.e., state or county) *i* as the ratio of mobilization size $M_{i}$ and population size $N_{i}$ as

1 $$\begin{aligned} m_{i} = \frac{M_{i}}{N_{i}}. \end{aligned}$$

Figure 2a, b illustrate mobilizability (i.e., mobilization per capita) in each US county for different levels of political polarization. Under full polarization, counties in the East Coast which are politically and geographically closest to the origin are the most mobilized, while counties in the middle of the country are the least mobilized. We also observe strong heterogeneity across the US. On the contrary, mobilization has less heterogeneity across counties under no polarization. While the counties in the East Coast remain highly mobilized, the difference from less mobilized counties is reduced.

We further characterize this polarization effect by measuring the mobilization size and the heterogeneity for different levels of polarization. In Fig. 2c, d, the total size of mobilization decreases by roughly 50% as polarization increases from 0 (i.e., no polarization) to 1 (i.e, full polarization) consistent with the overall patterns in Fig. 2a, b. The total size of mobilization is comparable to the number of signatures required for a petition to the White House (i.e., 100k).

In addition to the mobilization size, we measure the heterogeneity of mobilizability across the US counties using the Gini coefficient which is mostly used for economic inequality and ranging from 0 (complete equality) to 1 (complete inequality). As a result, increasing polarization leads to an increasing Gini coefficient from around 0.5 to 0.7. This result is also consistent with the high heterogeneity under full polarization in Fig. 2a. Thus, political polarization impedes overall mobilization with increasing the gap between regions by breaking recruitment between oppositely polarized individuals. This reachability gap by polarization makes a critical difference between states and counties when mobilized individuals push for coordinated action for trusting or distrusting information and policies.

## A hypothetical compliance campaign by mobilization

To demonstrate how mobilization promotes the very-early responses to the pandemic, we simulate a hypothetical compliance campaign driven by activists who would make calls to politicians or petition for precautionary measures. By doing so, we show how political polarization and the political landscape affect success of the campaign. This campaign is assumed to start on March 11 (i.e., the day of WHO pandemic declaration), and is marked as successful when a certain fraction of the state population is mobilized. Then, we compare the date of success with the actual date of lockdowns to show the potential of the campaign as a very-early warning method to mitigate the pandemic.

**Fig. 3:**
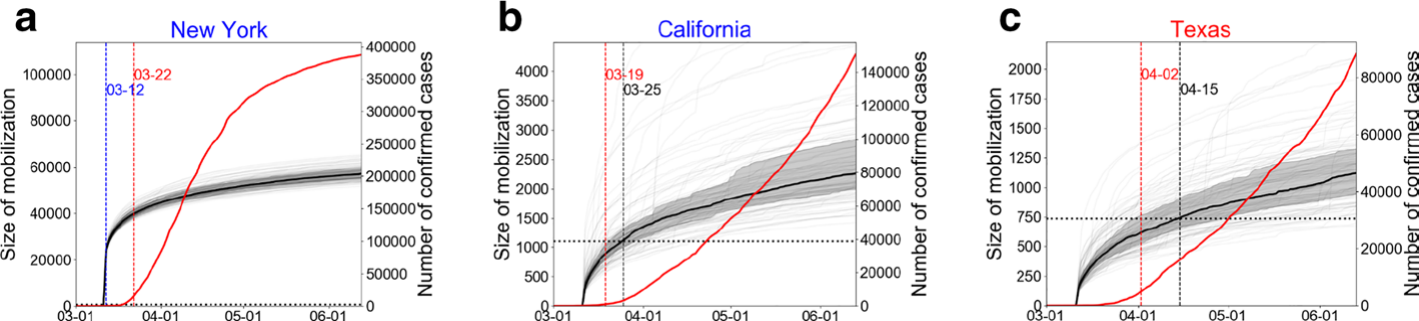
Time series of the mobilization size in states in a hypothetical compliance campaign: the case of **a** New York, **b** California, and **c** Texas. The campaign is assumed to start from New York City on March 11. The solid black line is the median time series of 100 simulations denoted by transparent lines. The interquartile range of 100 simulations is denoted by grey shades. The red curve shows the cumulative number of confirmed cases as recorded in The New York Times, based on reports from state and local health agencies. The dates of actual lockdowns are denoted by vertical red dotted lines, and the dates when the mobilization size of the median time series reaches 0.003% of the state population is denoted by blue (earlier than lockdowns) or black (later than lockdowns) dotted lines. See Additional file 1 for the result for all states

For a campaign started from Democratic seeds in New York City, NY, hit by the earliest surge of COVID-19, the simulations show growth of mobilization in each state with sharp growth at the beginning of the campaign and the following gradual increase (see Fig. 3). The earlier growth pattern was driven by seeding as it appears in the duration of the first few days comparable to the mean activation time of seeds (i.e., 1.5 days). A mobilization process essentially slows down as this mean activation time increases (see Additional file 1 for the sensitivity analysis). After this seeding phase, friendship-based mobilization retards the growth rate as the number of recruited friends decays over generations given by the mean branching factor less than one (i.e., $\langle k \rangle < 1$). The overall growth pattern is robust for seeding from Seattle, WA, another city hit by the earliest surge of COVID-19 (see Additional file 1).

**Fig. 4:**
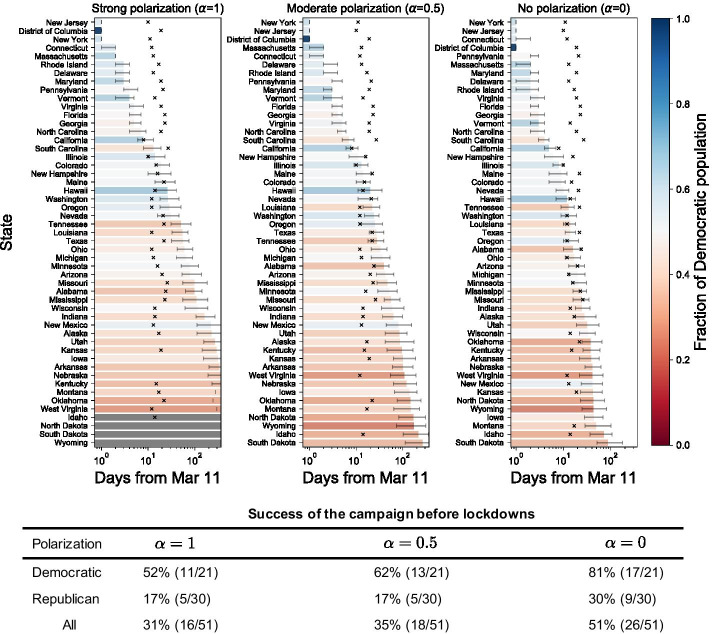
Days to success of compliance campaigns (bar plot) under different levels of polarization: strong polarization ($\alpha = 1$), moderate polarization ($\alpha = 0.5$), and no polarization ($\alpha = 0$). We seed the campaign from a 50,000 Democratic population in New York City (i.e., Kings county, NY) on March 11, 2020. We used the median time series of 100 simulations to determine the date of success. The bars denote the days taken to mobilize 0.003% of the population of each state in a span of one year, and the colors denote the political makeup of each state. States are colored by grey if mobilization failed to reach the population threshold. The error bars show the days when the upper and lower limits of the interquartile range (Fig. 3) of the mobilization sizes reach the population threshold. The cross markers denote the date of actual lockdown in each US state. The table under the plot shows the success rate of the campaign and the number of mobilized states for Democratic, Republican and all states, respectively

Using these growth patterns of mobilization, we estimate the expected date of success of mobilization for each state, and see if it gets ahead of actual lockdowns. We take the median values of 100 simulations for each day, and reconstruct a single median curve for each state. A campaign is marked as success when the mobilization size of this median curve exceeds a certain fraction (i.e., 0.003%) of the state population. This threshold was chosen to capture meaningful differences between states in a few weeks after the pandemic declaration.

Figure 4 shows earlier success of the compliance campaign in Democratic states. In general, blue states have earlier success than red states regardless of the degree of polarization. Our simulations estimate the success of the promotion before the dates of actual lockdowns in 52% of Democratic states in contrast to a significantly lower success rate of 17% in Republican states (Fig. 4a). The degree of political polarization not only impedes the overall success rate but also differentiates Democratic and Republican states. As polarization increases from 0 to 1, the number of states in which the campaign has succeeded before lockdowns decreases from 26 states to 16 states for seeding from New York City, and from 38 states to 16 states for seeding from Seattle (see Additional file 1). This result gives insights on how a gap between political parties can be linked to the diffusion of behavioral changes among the public. For example, a huge gap on precautionary measures can be interpreted as a high degree of polarization, which makes it difficult for the public to accept mobilization or ideas from oppositely polarized peers. The increased degree of polarization leads to slowed growth of mobilization and a critical difference between states in our model.

**Fig. 5:**
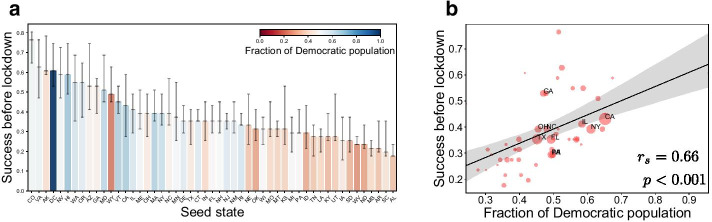
Success of the campaign by different seed states. **a** The ratio of states in which a compliance campaign succeeded mobilization before lockdowns for a given seed state under moderate polarization ($\alpha =0.5$). The most populated county of each seed state is chosen as the seed county. We used the median time series of 100 simulations to determine the date of success. The error bars denote the success rate calculated for the upper and lower limits of the interquartile range of the mobilization sizes instead of the median value. **b** The rank correlation (i.e., $r_{s} = 0.66$) between the fraction of Democratic population and the success of campaign in each state. Each bullet denotes each state and is sized by the population

The different success rates of campaigns seeded from New York City and Seattle leave us questions about the role of seed states: which state is the best state for seeding the campaign and why some states are better for seeding? To answer this, we simulate the compliance campaign for each different seed state. Figure 5a shows that the campaigns seeded from Democratic states mobilize more states before the actual date of lockdowns in general: for example, Colorado is the best seed state while Alabama is the worst seed state. As the seeds are assumed to be Democratic in the simulations, people are mobilized more rapidly in Democratic seed states, and further recruit their friends through the friendship network. Figure 5b confirms the correlation between the political makeup and the success of the campaign by seed states with a high correlation $r_{s} = 0.66$. This result is robust for different seeding locations where seeds are distributed to every county of a state in proportion to its Democratic or Republican population (see Additional file 1). Therefore, the simulations for all seed states confirm this biased influence spreading by political polarization.

**Fig. 6:**
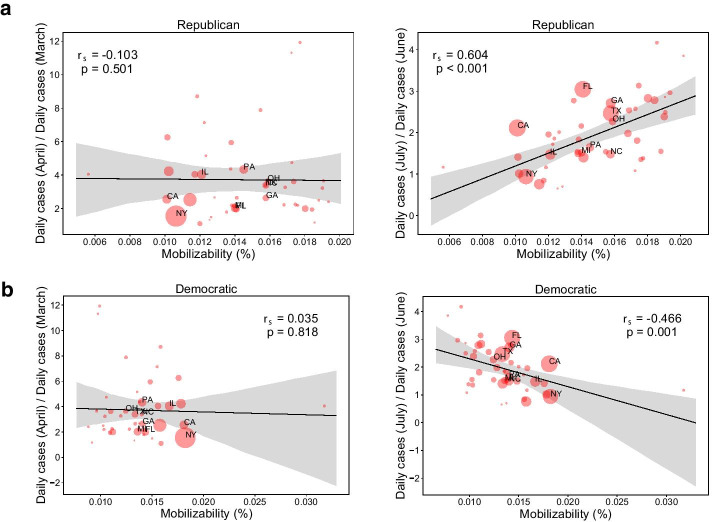
The coupling of mobilizability and growth of COVID-19. The rank correlation between the mobilizability and the growth rate of infection in states between March and April (left), and between June and July (right) for a Republican-oriented campaign (**a**) and Democratic-oriented campaign (**b**). We measure the mobilizability of each state in the simulation on the 7th day from the beginning. The mobilization size is estimated from the median time series of 100 simulations as in Fig. 3. The growth rate compares the average daily new confirmed cases between two consecutive months. A few states with a small number of confirmed cases less than 1000 on June 1st (i.e., Alaska, Hawaii, Montana, Vermont and Wyoming) are excluded in the correlation

## Coupling of mobilization and the growth of COVID-19

So far, we have examined how a compliance campaign from a county with the earliest surge could mobilize the other states for an early warning. Then, can we observe any coupling of these mobilization processes and the spread of COVID-19? If this coupling exists, it suggests that mobilization could have been related to mitigation or even intensification of the epidemics.

Here, we identify this coupling by comparing the mobilization size in our simulation with the growth of COVID-19. To demonstrate a situation that individuals are voluntarily mobilized in every state, we assume that there are 50,000 seeds distributed to every county of the US in proportion to its Democratic or Republican population according to the political orientation of mobilization (i.e., Democratic-oriented or Republican oriented). Then, the mobilizability of each state in the simulation is compared with the monthly growth rate of COVID-19 which is the ratio of the average daily new confirmed cases between two consecutive months.

As a result, we observe that the mobilizability from Republican populations is increasingly correlated with the growth rate of COVID-19 from the negligible correlation in March to $r_{s}$ = 0.60 in July, while the mobilizability from Democratic populations is negatively correlated to $r_{s}$ = -0.47 (see Fig. 6, and see Additional file 1 for the correlations in each month). This result corresponds to three different phases of polarized mobilization during the pandemic: (1) voluntary self-quarantine by Democrats from March, (2) anti-lockdown protests by Republicans from mid-April, and (3) Black Lives Matter protests led by Democrats from late May. The self-quarantine and anti-lockdown protests appear to have relatively increased the growth rate of infection in Republican states, while Black Lives Matter protests seem to have not made the opposite effect. These findings show a probable interconnection of mobilization and epidemics with an early warning of anti-lockdown protests. In this case, increased lockdowns in response to a second-wave could exacerbate the outbreak in contrary to its intention.

## Discussion

Social mobilization helps to understand collective responses, mediated by social influence, to the spread of COVID-19 and the countermeasures. Political polarization may impede the overall spread of social influence through the social network by inhibiting mobilization between oppositely polarized individuals. According to this homophilic mobilization, our hypothetical compliance campaign demonstrates that the political landscape controls mobilization, showing a higher success rate in non-polarized regions. The increasing coupling of mobilizability and growth of COVID-19 cases is consistent with a probable connection between a series of mobilization-driven movements and the epidemic spreading.

Our findings give us a few lessons for non-pharmaceutical interventions. Political polarization can be a double-edged sword for mitigation of COVID-19, yet it is more likely to be a net risk. In general, polarization works as friction or drag force against any type of mobilization or campaigns, so it can inhibit both anti-virus measures and anti-measure campaigns. Nevertheless, as NPIs are intended for coordinated action, political polarization is more likely to incapacitate the efforts to mitigate the spread. Emerging polarization after the pandemic [35] can create significant hurdles against pandemic control. Therefore, we suggest political efforts to bypass and/or alleviate political polarization for better efficacy of NPIs. For example, it would be effective to seed “sponsors” or “proponents” of promoting masks in oppositely oriented places to accomplish the promotion without passing through a polarization barrier .

Networked mobilization has a potential as a collective early-warning mechanism for pandemic control. Our hypothetical compliance campaign shows a potential of a mobilization-based warning that might have drastically mitigated the outbreak by earlier interventions. Again, this finding highlights that understanding the loss of efficacy by polarization is essential before implementing this effort. For practical applications, promotion of these mobilization campaigns and establishment of monitoring systems would help better responses to the next waves.

In addition, we should preemptively understand the nature, parameters and unintended effects of polarization before they are required to be factored into epidemic containment measures. Polarization is complex and can exist in many different dimensions such as economic, cultural, political and demographic dimensions. Social mobilization on social networks would face unintended effects in these dimensions, e.g., unexpected failure of campaigns, as has been observed in few open social challenges. We therefore call for thorough testing of mobilization as a phenomenon before it is required (Rutherford et al. 2020). Further, governments, societies and individuals must carefully weigh the tradeoffs of using social mobilization to exert coordinated action against the pandemic.

Lastly, this study is not without limitations. Although polarized mobilization in our simulation is correlated with the spreads of COVID-19, it does not provide any causal evidence. Further behavioral and epidemiological studies are needed to find out the causal relationship. In addition, our model focused only on political polarization, and hence it cannot explain the effects by other social layers such as income, gender and race, and complex behaviors such as spillover by lockdowns (Holtz et al. 2020), rising protests and the surge during summer holidays. Fortunately, the friendship network based on the Facebook dataset can be considered as a ground-truth of social connections (Bailey et al. 2018), so it contains the aggregated information of sociodemographic variables, despite a potential bias to a younger population with more technical backgrounds. Recent behavioral studies on political partisanship and COVID-19 (Gollwitzer et al. 2020; Grossman et al. 2020) also highlight politics as a major determinant of behavior as well as showing consistency with our observations. Thus, our fundamental model provides a theoretical background for the diffusion of behavioral changes modulated by political polarization. Finally, we remark that adversarial behavior could disturb mobilization processes. Indeed, we have observed several intended and unintended adversarial activities against mobilization during the pandemic, for example, asserting uselessness of wearing face masks. As the impact of adversarial activities on polarized mobilization is hardly predictable, it requires further theoretical and data-driven studies.

## Supplementary Information

**Additional file 1**. Supplementary information for hypothetical compliance campaigns (Section S1) and the coupling of mobilization and the growth of COVID-19 (Section S2).

## Data Availability

The US county-wise friendship data may be available for download from Facebook upon request. $\bullet$ https://dataforgood.fb.com/tools/social-connectedness-index. The voting records in the 2016 US Presidential Election were downloaded from Opendatasoft which collected the raw records from The New York Times. $\bullet$ Opendatasoft https://public.opendatasoft.com/explore/dataset/usa-2016-presidential-election-by-county $\bullet$ The New York Times https://www.nytimes.com/elections/2016/results/president $\bullet$ The demographics of counties are available from the US Census Bureau. https://data.census.gov/cedsci/. The COVID-19 data in the United States based on reports from state and local health agencies is available for download from The New York Times. $\bullet$ https://github.com/nytimes/covid-19-data.

## Abbreviations

COVID-19: Coronavirus disease 2019
NPIs: Non-pharmaceutical interventions
SCI: Social Connectedness Index
WHO: World Health Organization

## References

[CR1] Aleta A, Martín-Corral D, y Piontti AP, Ajelli M, Litvinova M, Chinazzi M, Dean NE, Halloran ME, Longini Jr IM, Merler S et al (2020) Modelling the impact of testing, contact tracing and household quarantine on second waves of covid-19. Nat Hum Behav 1–8 10.1038/s41562-020-0931-9 PMC7641501 32759985

[CR2] Alstott J, Madnick S, Velu C (2014). Homophily and the speed of social mobilization: the effect of acquired and ascribed traits. PLoS ONE.

[CR3] Bailey M, Cao R, Kuchler T, Stroebel J, Wong A (2018). Social connectedness: measurement, determinants, and effects. J Econ Perspect.

[CR4] Bakshy E, Messing S, Adamic LA (2015). Exposure to ideologically diverse news and opinion on facebook. Science.

[CR5] Bento AI, Nguyen T, Wing C, Lozano-Rojas F, Ahn Y-Y, Simon K (2020). Evidence from internet search data shows information-seeking responses to news of local covid-19 cases. Proc Natl Acad Sci.

[CR6] Benzell SG, Collis A, Nicolaides C (2020) Rationing social contact during the covid-19 pandemic: transmission risk and social benefits of us locations. Proc Natl Acad Sci 10.1073/pnas.2008025117 PMC7334565 32522870

[CR7] Cebrian M, Coviello L, Vattani A, Voulgaris P (2012) Finding red balloons with split contracts: robustness to individuals’ selfishness. In: Proceedings of the forty-fourth annual ACM symposium on theory of computing, pp 775–788

[CR8] Chen H, Rahwan I, Cebrian M (2016). Bandit strategies in social search: the case of the DARPA red balloon challenge. EPJ Data Sci.

[CR9] Chinazzi M, Davis JT, Ajelli M, Gioannini C, Litvinova M, Merler S, y Piontti AP, Mu K, Rossi L, Sun K (2020). The effect of travel restrictions on the spread of the 2019 novel coronavirus (covid-19) outbreak. Science.

[CR10] Cruickshank IJ, Carley KM (2020). Characterizing communities of hashtag usage on twitter during the 2020 covid-19 pandemic by multi-view clustering. Appl Netw Sci.

[CR11] Desvars-Larrive A, Dervic E, Haug N, Niederkrotenthaler T, Chen J, Di Natale A, Lasser J, Gliga DS, Roux A, Chakraborty A, Ten A, Dervic A, Pacheco A, Cserjan D, Lederhilger D, Berishaj D, Flores Tames E, Takriti H, Korbel J, Reddish J, Stangl J, Hadziavdic L, Stoeger L, Gooriah L, Geyrhofer L, Ferreira MR, Vierlinger R, Holder S, Alvarez S, Haberfellner S, Ahne V, Reisch V, Servedio VD, Chen X, Pocasangre-Orellana XM, Garcia D, Thurner S (2020). A structured open dataset of government interventions in response to covid-19. Sci Data.

[CR12] Epstein Z, Epstein M, Almenar C, Groh M, Pescetelli N, Moro E, Obradovich N, Cebrian M, Rahwan I (2019) Towards a new social laboratory: an experimental study of search through community participation at burning man. arXiv preprint arXiv:1903.04125

[CR13] Gollwitzer A, Martel C, Brady WJ, Pärnamets P, Freedman IG, Knowles ED, Van Bavel JJ (2020) Partisan differences in physical distancing are linked to health outcomes during the covid-19 pandemic. Nat Hum Behav 1–12 10.1038/s41562-020-00977-7 33139897

[CR14] Grossman G, Kim S, Rexer JM, Thirumurthy H (2020). Political partisanship influences behavioral responses to governors’ recommendations for covid-19 prevention in the United States. Proc Natl Acad Sci.

[CR15] Han J, Cha M, Lee W (2020) Anger contributes to the spread of covid-19 misinformation. Harvard Kennedy Sch Misinf Rev 1(3)

[CR16] Holtz D, Zhao M, Benzell SG, Cao CY, Rahimian MA, Yang J, Allen J, Collis A, Moehring A, Sowrirajan T, Ghosh D, Zhang Y, Dhillon PS, Nicolaides C, Eckles D, Aral S (2020). Interdependence and the cost of uncoordinated responses to covid-19. Proc Natl Acad Sci.

[CR17] Iribarren JL, Moro E (2009). Impact of human activity patterns on the dynamics of information diffusion. Phys Rev Lett.

[CR18] Johnson NF, Velásquez N, Restrepo NJ, Leahy R, Gabriel N, El Oud S, Zheng M, Manrique P, Wuchty S, Lupu Y (2020) The online competition between pro-and anti-vaccination views. Nature 1–4 10.1038/s41586-020-2281-1 32499650

[CR19] Kim H, Walker D (2020) Leveraging volunteer fact checking to identify misinformation about covid-19 in social media. Harvard Kennedy Sch Misinf Rev 1(3)

[CR20] Kraemer MU, Yang C-H, Gutierrez B, Wu C-H, Klein B, Pigott DM, Du Plessis L, Faria NR, Li R, Hanage WP (2020). The effect of human mobility and control measures on the covid-19 epidemic in China. Science.

[CR21] Naroditskiy V, Rahwan I, Cebrian M, Jennings NR (2012). Verification in referral-based crowdsourcing. PLoS ONE.

[CR22] Naroditskiy V, Jennings NR, Van Hentenryck P, Cebrian M (2014). Crowdsourcing contest dilemma. J R Soc Interface.

[CR23] Oishi K, Cebrian M, Abeliuk A, Masuda N (2014). Iterated crowdsourcing dilemma game. Sci Rep.

[CR24] Pickard G, Pan W, Rahwan I, Cebrian M, Crane R, Madan A, Pentland A (2011). Time-critical social mobilization. Science.

[CR25] Rahwan I, Dsouza S, Rutherford A, Naroditskiy V, McInerney J, Venanzi M, Jennings NR, Cebrian M (2012). Global manhunt pushes the limits of social mobilization. Computer.

[CR26] Republicans, Democrats Move Even Further Apart in Coronavirus Concerns. Pew Research Center. Accessed 2020-09-20

[CR27] Rutherford A, Cebrian M, Rahwan I, Dsouza S, McInerney J, Naroditskiy V, Venanzi M, Jennings NR, DeLara J, Wahlstedt E (2013). Targeted social mobilization in a global manhunt. PLoS ONE.

[CR28] Rutherford A, Cebrian M, Dsouza S, Moro E, Pentland A, Rahwan I (2013). Limits of social mobilization. Proc Natl Acad Sci.

[CR29] Rutherford A, Cebrian M, Hong I, Rahwan I (2020) Impossible by conventional means: ten years on from the DARPA red balloon challenge. arXiv preprint arXiv:2008.05940

[CR30] Sims MH, Bigham J, Kautz H, Halterman MW (2014). Crowdsourcing medical expertise in near real time. J Hosp Med.

[CR31] Stefanovitch N, Alshamsi A, Cebrian M, Rahwan I (2014). Error and attack tolerance of collective problem solving: The DARPA Shredder Challenge. EPJ Data Sci.

[CR32] Stephen AT, Lehmann DR (2016). How word-of-mouth transmission encouragement affects consumers’ transmission decisions, receiver selection, and diffusion speed. Int J Res Mark.

[CR33] Tang JC, Cebrian M, Giacobe NA, Kim H-W, Kim T, Wickert DB (2011). Reflecting on the DARPA Red Balloon Challenge. Commun ACM.

[CR34] The Quiet Hand of Conservative Groups in the Anti-Lockdown Protests. The New York Times. Accessed 2020-09-16

[CR35] Van Bavel JJ, Baicker K, Boggio PS, Capraro V, Cichocka A, Cikara M, Crockett MJ, Crum AJ, Douglas KM, Druckman JN et al (2020) Using social and behavioural science to support covid-19 pandemic response. Nat Hum Behav 1–12 10.1038/s41562-020-0884-z 32355299

[CR36] Wang J, Madnick S, Li X, Alstott J, Velu C (2015). Effect of media usage selection on social mobilization speed: Facebook vs e-mail. PLoS ONE.

[CR37] Why some protesters in America wear Hawaiian shirts. The Economist. Accessed 2020-09-16

