# Supplementary material for: Social mobilization and polarization can create volatility in COVID-19 pandemic control
Source: Appl Netw Sci. 2021 Feb 11;6(1):11. doi: 10.1007/s41109-021-00356-9 (PMC7877319; doi:10.1007/s41109-021-00356-9)
Supplement: Supplementary file 1 — Additional file 1. Supplementary information for hypothetical compliance campaigns (Section S1) and the coupling of mobilization and the growth of COVID-19 (Section S2). [file 41109_2021_356_MOESM1_ESM.pdf]

Supplementary Information

S1 Hypothetical compliance campaign

S1.1 Time series of the mobilization size in each state

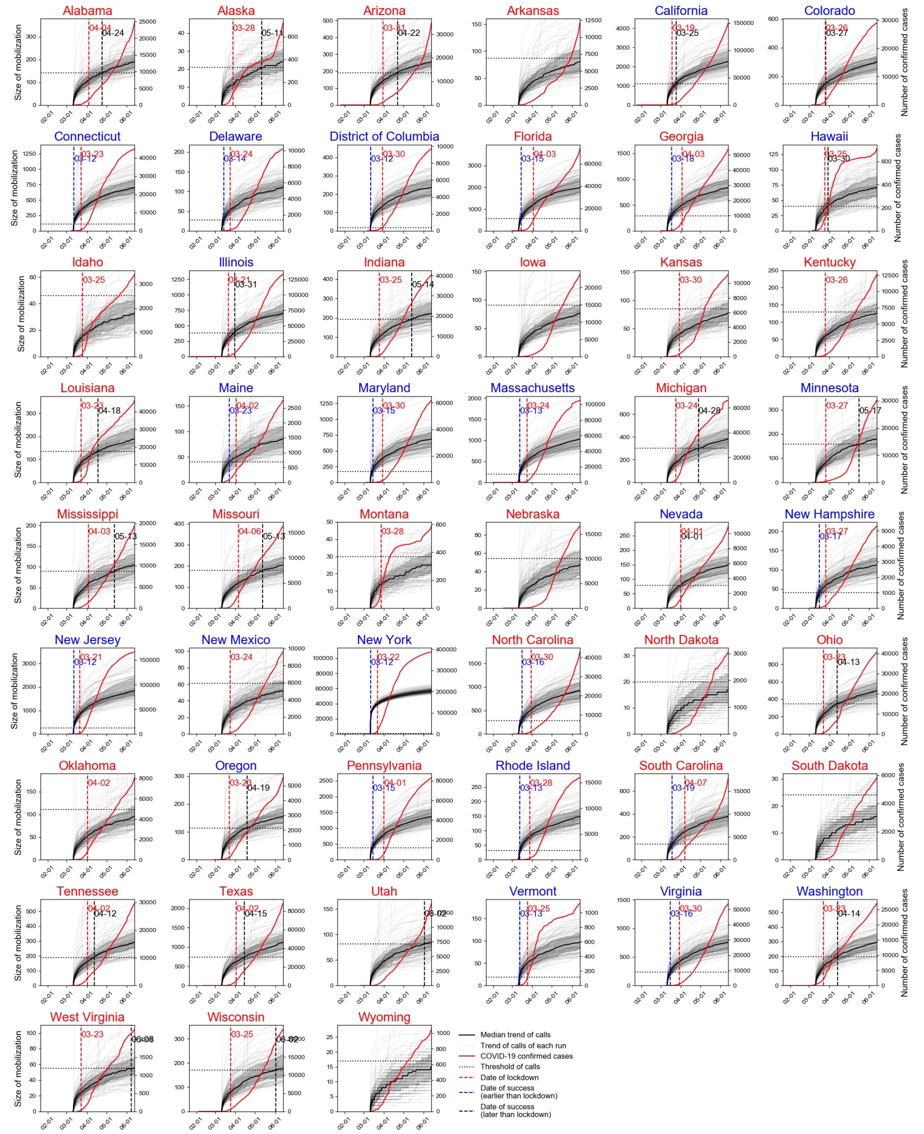

Figure S1 Time series of the mobilization size in each state for the simulation started from Democratic populations in New York City. The campaign is assumed to start from New York City on March 11. The dotted line is the median time series of 100 simulations denoted by transparent lines. The dates of actual lockdowns are denoted by vertical red dotted lines, and the dates when the mobilization size of the median time series reaches 0.003% of the state population is denoted by blue (earlier than lockdowns) or black (later than lockdowns) dotted lines.

Figure S1 shows the time series of the mobilization size in each state for the simulation seeded from Kings County, NY under intermediate polarization ($\alpha = 0.5$). The mobilization size sharply grows at the beginning of the campaign and gradually increases after that, showing consistency across states.

S1.2 Seeding from Seattle, WA

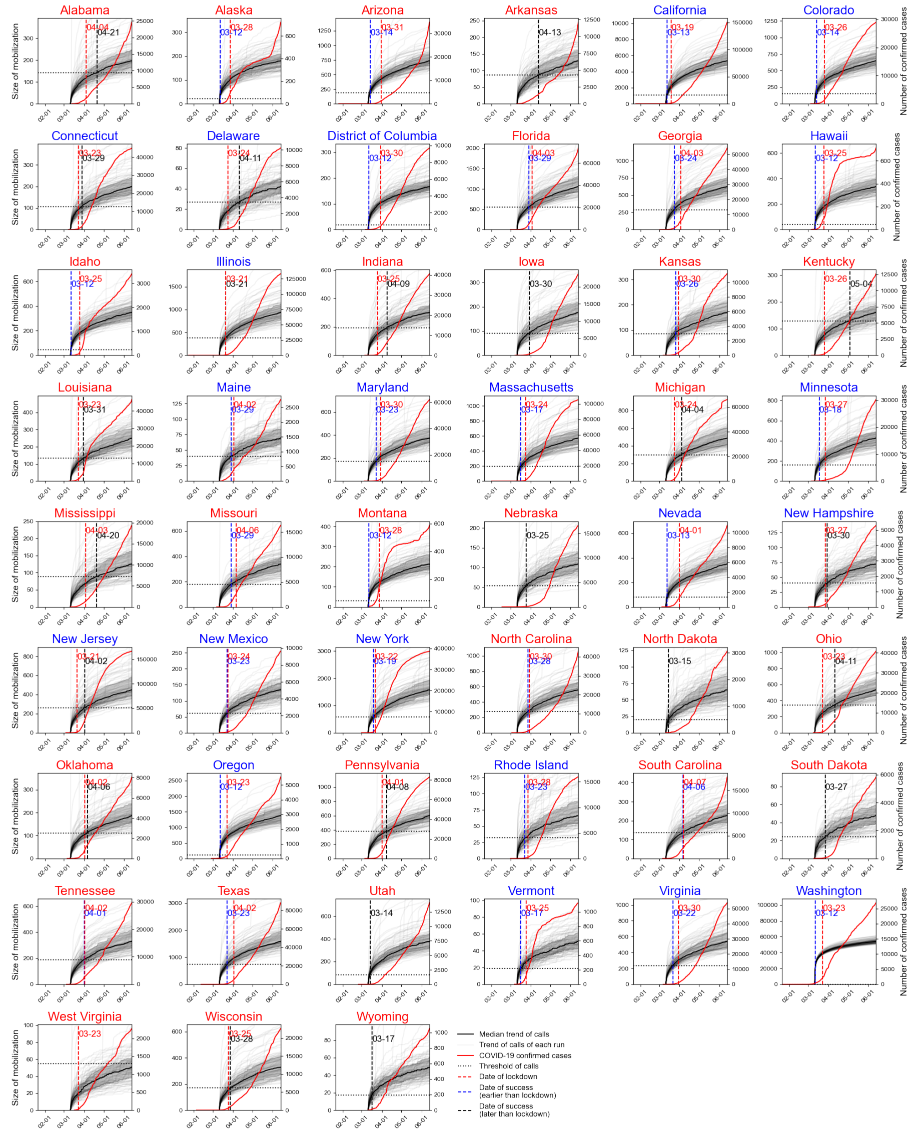

Figure S2 Time series of the mobilization size in each state for the simulation started from Democratic populations in Seattle. All the parameters and descriptions are the same with Figure S1.

Seattle is one of the cities hit by the earliest surge of COVID-19. We repeated the simulation for a campaign seeded from the most populated county (i.e., King County, WA) of Seattle. As a result, we observe a similar pattern with the simulation seeded from New York. Figure S2 reproduces the curve of the mobilization size with rapid growth at the beginning of the campaign and the following gradual increase. Figure S3 also has a similar pattern with the simulation seeded from New York, showing a higher success rate of a Democratic-oriented campaign in Democratic states. The success rate also decreases with increasing political polarization.

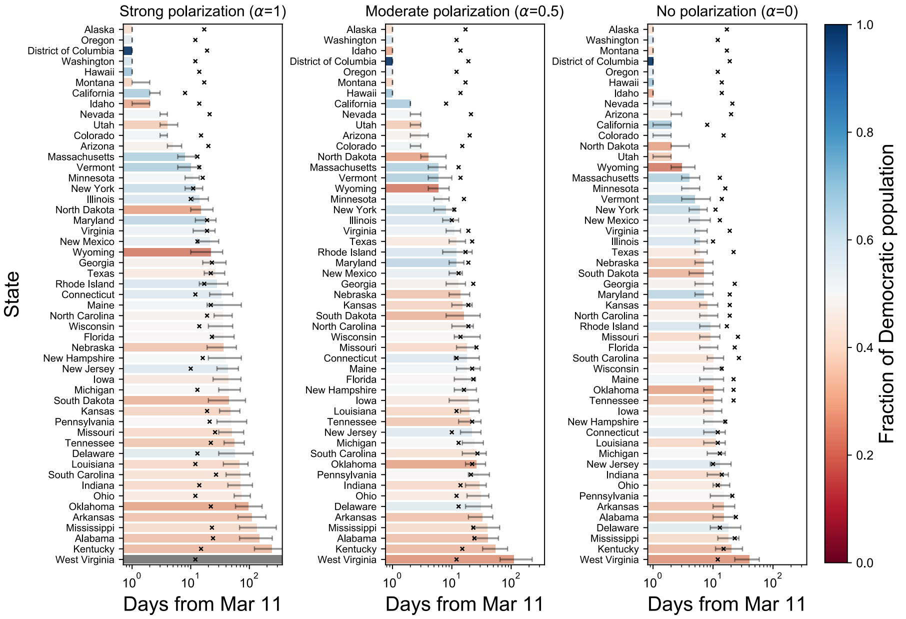

Success of the campaign before lockdowns

Polarization	$\alpha = 1$	$\alpha = 0.5$	$\alpha = 0$
Democratic	57% (12/21)	76% (16/21)	90% (19/21)
Republican	13% (4/30)	40% (12/30)	63% (19/30)
All	31% (16/51)	55% (28/51)	75% (38/51)

Figure S3 Days to success of compliance campaigns (bar plot) seeded from Seattle, WA under different levels of polarization: (a) strong polarization ($\alpha = 1$), (b) moderate polarization ($\alpha = 0.5$), and (c) no polarization ($\alpha = 0$). The seeds are all Democratic. The bars show the days taken to mobilize 0.003% of the population of each state, and the colors denote the political makeup of each state. The cross markers denote the date of actual lockdown in each US state.

S1.3 Seeding from all counties in a state

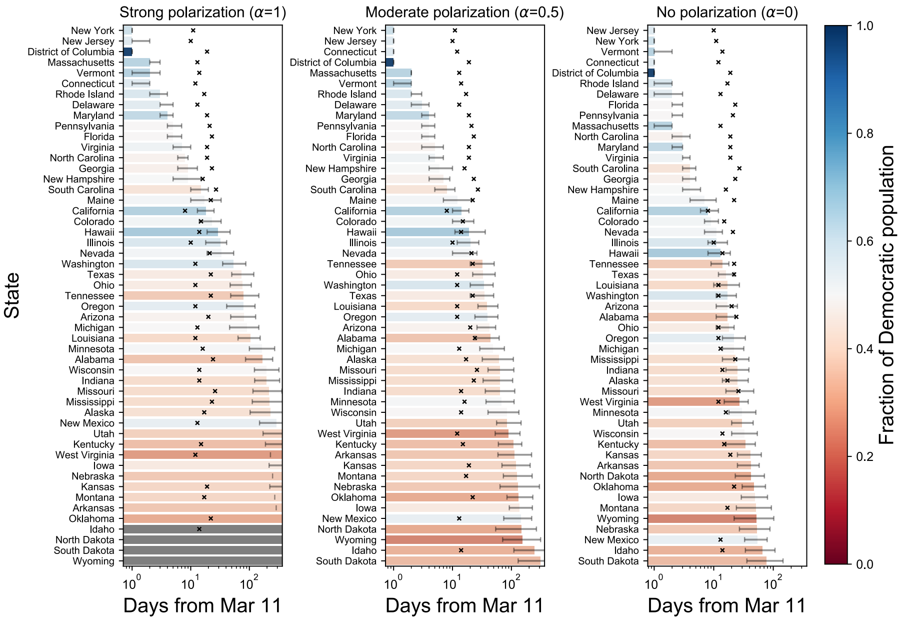

Success of the campaign before lockdowns

Polarization	$\alpha = 1$	$\alpha = 0.5$	$\alpha = 0$
Democratic	57% (12/21)	57% (12/21)	71% (17/21)
Republican	17% (5/30)	17% (5/30)	30% (11/30)
All	33% (17/51)	33% (17/51)	47% (28/51)

Figure S4 Days to success of compliance campaigns (bar plot) seeded from all counties in New York under different levels of polarization: (a) strong polarization ($\alpha = 1$), (b) moderate polarization ($\alpha = 0.5$), and (c) no polarization ($\alpha = 0$). The seeds are all Democratic, and are distributed to every county in proportion to its Democratic population. The bars show the days taken to mobilize 0.003% of the population of each state, and the colors denote the political makeup of each state. The cross markers denote the date of actual lockdown in each US state.

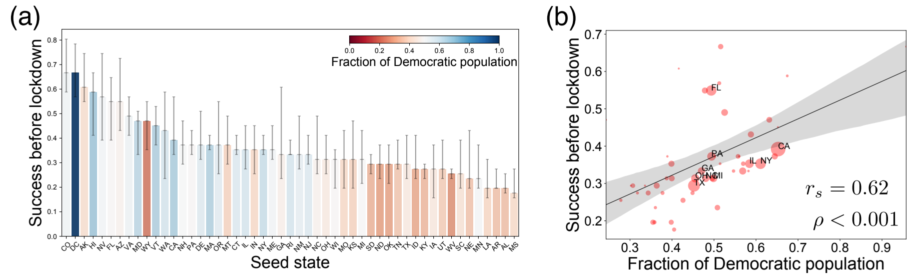

Figure S5 Success of the campaign by different seed states. (a) The ratio of states in which a compliance campaign succeeded mobilization before lockdowns for a given seed state under moderate polarization ($\alpha = 0.5$). We used the median time series of 100 simulations to determine the date of success. 50,000 Democratic seeds are distributed to every county of a seed state in proportion to the county's Democratic population. (b) The rank correlation (i.e., $r_s = 0.62$) between the fraction of Democratic population and the success of campaign in each state. Each state is denoted by each bullet and sized by its population.

In most of simulations, we used seeding from the most populated county of a state as default. This setup could make a bias toward Democratic populations since large counties generally have a higher Democratic proportion compared to rural counties in the same state. Here, we check the robustness of the results for seeding methods. Specifically, we test a seeding method where seeds are distributed to every county of a state in proportion to the county's Democratic or Republican population.

Figure S4 shows the date of success for seeding from all counties in NY. It shows a very similar pattern – mobilization impeded by polarization – with the result from our previous seeding method based on the most populated county. Likewise, we check the consistency of the success rate of the campaign for different seed states in Figure S5. It is also consistent with our previous result that showed a higher success rate in Democratic states for Democratic-oriented mobilization. Therefore, the results are robust for seed locations in the same state. It seems that similar structures of the friendship network over different counties in a state due to the geographical proximity [1] makes little differences by seeding methods.

S1.4 Sensitivity analysis

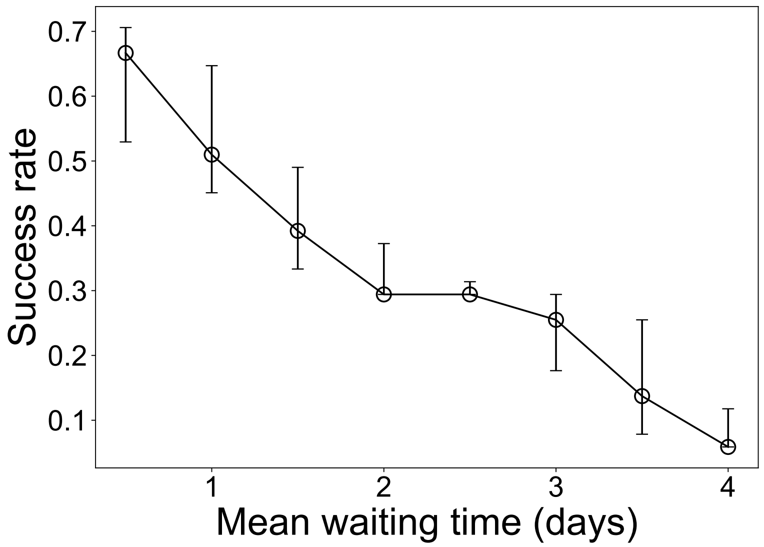

Figure S6 The success rate of a campaign by different mean waiting times. The seeds are all Democratic, and are located in the most populated county (i.e., Kings County, NY) in New York City. The success rate is the proportion of states where the mobilization size exceeds 0.003% of the state population before its actual date of lockdown. The error bars denote the success rate calculated for the upper and lower limits of the interquartile range of the mobilization sizes instead of the median value.

In this section, we examine the sensitivity of a hypothetical compliance campaign for different waiting times of activation. In each simulation step, a recruited individual is activated, and it further proceed mobilization. The activation time (i.e., waiting time) of each individual is determined by a log-normal distribution with a mean of 1.5 day and a standard deviation of 5.5 days following the information diffusion dynamics [2]. Here we test the sensitivity of our results for different mean waiting times ranging from 0.5 day to 4 days. As a result, Figure S6 shows a decreasing success rate as the mean waiting time increases. As the mean activation time determines the rate of activation of individuals in the queue, it is natural that the growth of mobilization slows down with an increasing activation time, and the mobilization process fails to reach the population threshold before the date of actual lockdown. Figure S7 shows the days to success in each state by different mean waiting times $\bar{t} = \{0.5, 1.5, 3.0\}$. The days to success are commonly delayed in most of states as the waiting time increases.

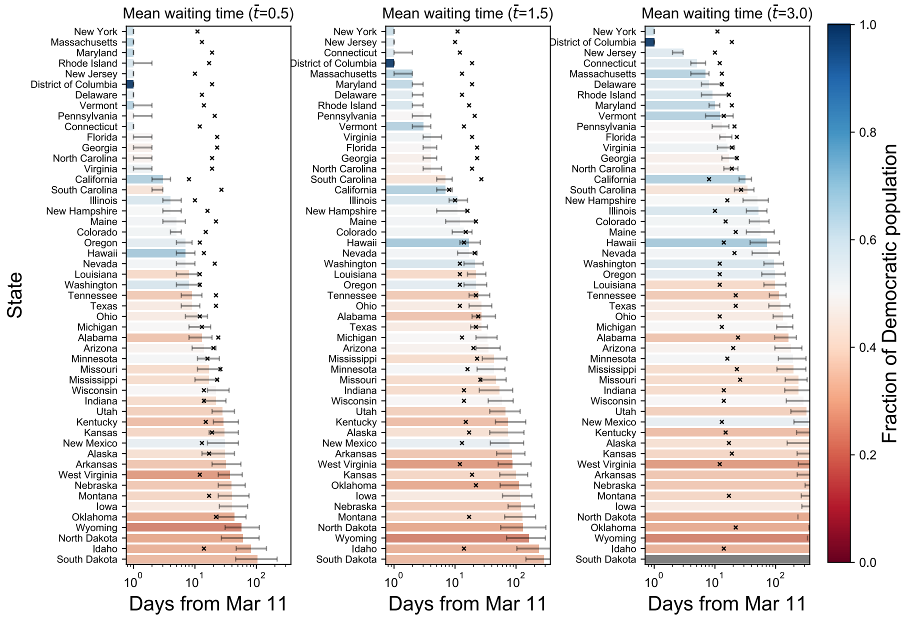

Success of the campaign before lockdowns

Mean waiting time	$\bar{t} = 0.5$	$\bar{t} = 1.5$	$\bar{t} = 3.0$
Democratic	95% (20/21)	71% (15/21)	48% (10/21)
Republican	47% (14/30)	17% (5/30)	10% (3/30)
All	67% (34/51)	39% (20/51)	25% (13/51)

Figure S7 Days to success of compliance campaigns (bar plot) seeded from New York City under different mean waiting times: (a) short waiting time ($\bar{t} = 0.5$), (b) intermediate waiting time ($\bar{t} = 1.5$), and (c) long waiting time ($\bar{t} = 3.0$). The seeds are all Democratic. The bars show the days taken to mobilize 0.003% of the population of each state, and the colors denote the political makeup of each state. The cross markers denote the date of actual lockdown in each US state.

S2 Coupling of mobilization and the growth of COVID-19

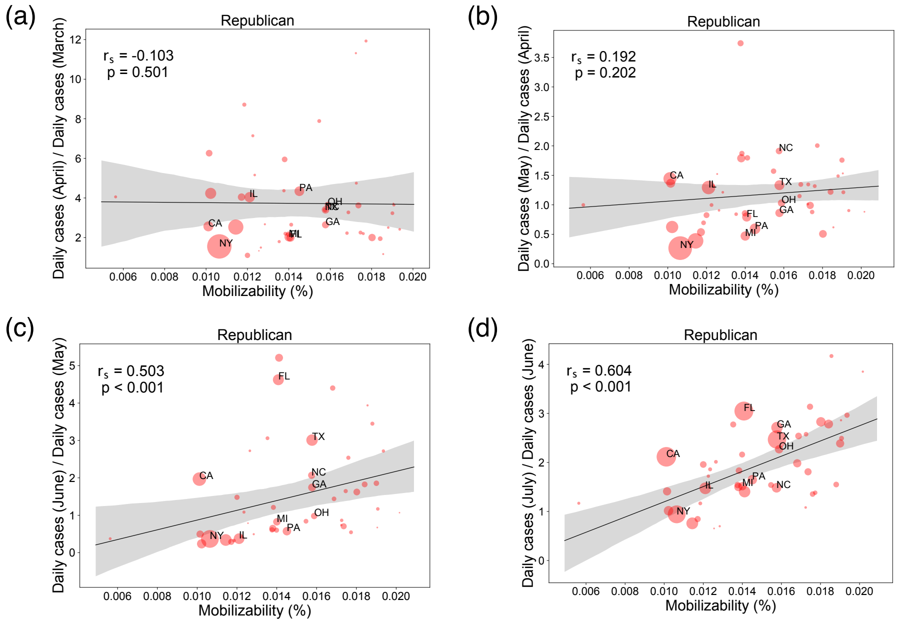

Figure S8 Correlation of Republican-driven mobilizability and growth of COVID-19 in April (a), May (b), June (c) and July (d) compared to the previous months.

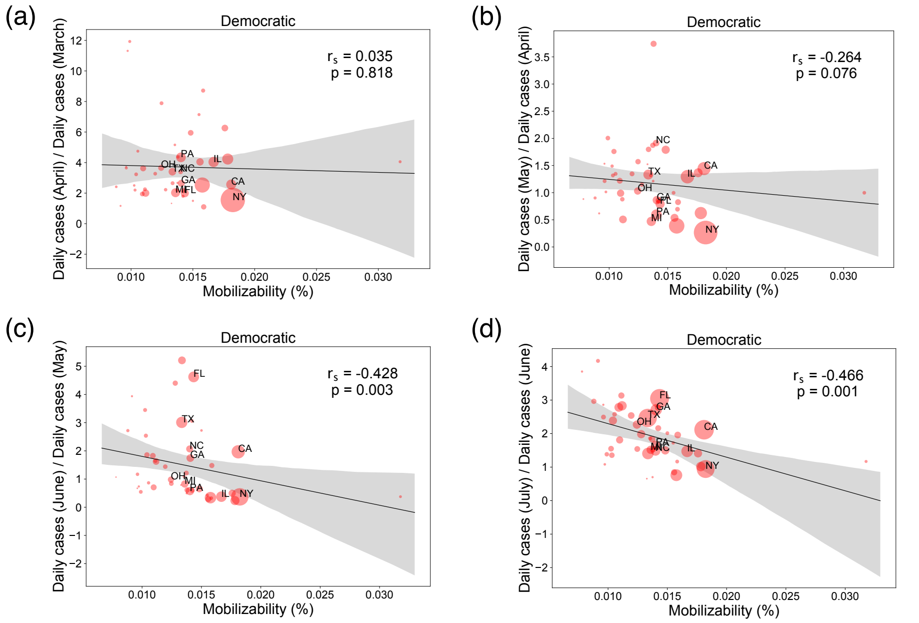

Figure S9 Correlation of Democratic-driven mobilizability and growth of COVID-19 in April (a), May (b), June (c) and July (d) compared to the previous months.

We identify the coupling of the mobilization size in our simulation and the growth of COVID-19. In this simulation, we assume that there are 50,000 seeds distributed

to every county in the US in proportion to the county's Democratic or Republican population according to the political orientation of mobilization. Then, we compared the mobilizability (i.e., the mobilization size divided by the state population) at the 7th day of the simulation and the monthly growth rate of the infected cases at the state level. As a result, we observe that the mobilizability from Republican populations is increasingly correlated with the growth rate of COVID-19 from the negligible correlation in March to r_s = 0.60 in July, while the mobilizability from Democratic populations is decreasingly correlated to r_s = -0.47.

References

1. Bailey, M., Cao, R., Kuchler, T., Stroebel, J., Wong, A.: Social connectedness: Measurement, determinants, and effects. Journal of Economic Perspectives **32**(3), 259–80 (2018)
2. Iribarren, J.L., Moro, E.: Impact of human activity patterns on the dynamics of information diffusion. Physical Review Letters **103**(3), 038702 (2009)
